# Aetiology of Heart Failure, Rather than Sex, Determines Reverse LV Remodelling Response to CRT

**DOI:** 10.3390/jcm10235513

**Published:** 2021-11-25

**Authors:** Fatema Said, Jozine M. ter Maaten, Pieter Martens, Kevin Vernooy, Mathias Meine, Cornelis P. Allaart, Bastiaan Geelhoed, Marc A. Vos, Maarten J. Cramer, Isabelle C. van Gelder, Wilfried Mullens, Michiel Rienstra, Alexander H. Maass

**Affiliations:** 1 Department of Cardiology, University Medical Center Groningen, University of Groningen, 9713 Groningen, The Netherlands; f.said@umcg.nl (F.S.); j.m.ter.maaten@umcg.nl (J.M.t.M.); b.geelhoed@umcg.nl (B.G.); i.c.van.gelder@umcg.nl (I.C.v.G.); m.rienstra@umcg.nl (M.R.); 2 Department of Cardiology, Ziekenhuis Oost-Limburg, 3600 Genk, Belgium; pieter_martens@icloud.com (P.M.); wilfried.mullens@gmail.com (W.M.); 3 Faculty of Medicine and Life Sciences, Biomedical Research Institute, Hasselt University, 3590 Diepenbeek, Belgium; 4 Department of Cardiology, Maastricht University Medical Center, 6200 Maastricht, The Netherlands; kevin.vernooy@mumc.nl; 5 Department of Cardiology, University Medical Center Utrecht, 3584 Utrecht, The Netherlands; m.meine@umcutrecht.nl (M.M.); m.j.m.cramer@umcutrecht.nl (M.J.C.); 6 Department of Cardiology, VU University Medical Center, 1081 Amsterdam, The Netherlands; cp.allaart@amsterdamumc.nl; 7 Department of Medical Physiology, University of Utrecht, 3584 Utrecht, The Netherlands; M.A.Vos@umcutrecht.nl

**Keywords:** cardiac resynchronization therapy, heart failure, sex differences

## Abstract

Introduction: Cardiac resynchronization therapy (CRT) is an established therapy for patients with heart failure with reduced ejection fraction (HFrEF). Women appear to respond differently to CRT, yet it remains unclear whether this is inherent to the female sex itself, or due to other patient characteristics. In this study, we aimed to investigate sex differences in response to CRT. Methods: This is a post-hoc analysis of a prospective, multicenter study (MARC) in the Netherlands, studying HFrEF patients with an indication for CRT according to the guidelines (*n* = 240). Primary outcome measures are left ventricular ejection fraction (LVEF) and left ventricular end systolic volume (LVESV) at 6 months follow-up. Results were validated in an independent retrospective Belgian cohort (*n* = 818). Results: In the MARC cohort 39% were women, and in the Belgian cohort 32% were women. In the MARC cohort, 70% of the women were responders (defined as >15% decrease in LVESV) at 6 months, compared to 55% of men (*p* = 0.040) (79% vs. 67% in the Belgian cohort, *p* = 0.002). Women showed a greater decrease in LVESV %, LVESV indexed to body surface area (BSA) %, and increase in LVEF (all *p* < 0.05). In regression analysis, after adjustment for BSA and etiology, female sex was no longer associated with change in LVESV % and LVESV indexed to BSA % and LVEF % (*p* > 0.05 for all). Results were comparable in the Belgian cohort. Conclusions: Women showed a greater echocardiographic response to CRT at 6 months follow-up. However, after adjustment for BSA and ischemic etiology, no differences were found in LV-function measures or survival, suggesting that non-ischemic etiology is responsible for greater response rates in women treated with CRT.

## 1. Introduction

Cardiac resynchronization therapy (CRT) is an established therapy for patients with heart failure with reduced ejection fraction (HFrEF) and ventricular conduction disturbance, which aims to improve cardiac function through resynchronizing electrical activation. CRT has been proven to be an effective treatment option as it improves quality of life, reduces heart failure (HF), hospitalization, and improves survival [1]. While numerous metrics exist to describe response to CRT, a striking observation is that not all patients exhibit the same left ventricular reverse remodeling response after implantation of CRT [2]. Several factors have been associated with different left ventricular reverse remodeling following CRT, including etiology of HF, QRS morphology, duration and area, and female sex [3,4,5,6,7]. Multiple studies have shown a greater response to CRT in women compared to men [6,8]. Women generally have a smaller body surface area (BSA), which may also be a driver for differences in response to heart failure therapy between women and men, reflected not only by different response to CRT treatment but for example also differences in optimal HF medication dosages [9]. It remains unclear whether the sex-related differences in the left ventricular reverse remodeling response to CRT is truly caused by female sex itself, or whether sex is a marker of another characteristic such as difference in BSA, LV size, or HF etiology [10,11]. We therefore aimed to investigate sex differences in reverse remodeling response to CRT using a thoroughly phenotyped, prospectively collected cohort (Markers and Response to CRT (MARC)), and validated this in a larger retrospective Belgian cohort. In order to test the accuracy of the findings of the index MARC cohort, a larger Belgian cohort was used as validation cohort. In addition, we aimed to study sex differences in other outcome measures to CRT as well, namely functional, clinical, and biochemical measures of response. We hypothesized that sex differences in response to CRT are mainly determined by the ischemic etiology of HF, classified by a medical history of myocardial infarction of revascularization.

## 2. Methods

### 2.1. Study Participant and Data Collection

The Markers and Response to CRT (MARC) study is a prospective, multicenter, observational study performed in the Netherlands, designed to identify a set of biomarkers that can predict the magnitude of reverse left ventricular (LV) remodeling after CRT. A total number of 240 patients were included in six participating centers between February 2012 and November 2013, all of which had a de novo indication for CRT according to the current American Heart Association (AHA) and European Society of Cardiology (ESC) guidelines [12,13]. This includes patients with a left bundle branch block (LBBB) and non-specific intraventricular conduction delay (IVCD), but without right bundle branch block, significant atrio-ventricular (AV) conduction delays, or recent episodes of atrial fibrillation (AF). Extensive data was collected in the MARC study, including clinical, electrocardiographic, and echocardiographic measures, as well as serum biomarkers were assessed. In both the MARC and the Belgian cohort, patients were categorized as having an ischemic cardiomyopathy if there was a history of myocardial infarction or revascularization [14]. All electrocardiographic and echocardiographic measures were assessed in the appropriate core lab for uniform and consistent assessments. Vectocardiographic measures (VCG) were synthesized from pre-procedural 12-leads electrocardiograms (ECGs). One month after implantation, all patients received echocardiographically guided optimization of atrio-ventricular and ventriculo-ventricular delays, as well as programming, of device settings to rate response unless good chronotropic response was observed at device check-up. More elaborate methods and the primary results of the MARC study have been published previously [5]. Data was collected at time of CRT implantation (baseline), 6 months, and 12 months. This study was approved by the institutional review boards of all participating centers (METc 2011/201, ethical approval received on 14 November 2011). Written consent has been given by all study participants.

For the Belgian cohort, consecutive patients with HFrEF undergoing CRT-implantation in compliance with the ESC guidelines in a single tertiary care center (Ziekenhuis Oost-Limburg, Genk, Belgium) between October 2008 and September 2016 were evaluated retrospectively. Unlike the MARC cohort, patients with AF were not excluded. Patients underwent a prespecified follow-up and post-CRT optimization protocol, which has been published previously [15]. In brief, all patients received standardized optimization of HF care and echocardiographically guided device optimization. Patients received their first follow-up at 6 weeks and the second follow-up at 6 months. Echocardiographic measures, as well as a cycloergometric bicycle test with maximum oxygen uptake (VO_2_ max) were assessed during the 6-month follow-up visit. Given the retrospective nature of the study design, the need for written informed consent was waived by the local ethics committee.

### 2.2. Response Measures

Our primary outcome measures were changes in echocardiographic markers of response, as well as biomarker and functional markers of response from baseline to 6 months after CRT implantation. More specifically, we studied absolute change and the % change in left ventricular ejection fraction (LVEF), left ventricular end systolic volume (LVESV), the % change in LVESV indexed to BSA, the number of responders (patients with >15% decrease in LVESV), the absolute and % change in N-terminal pro-B-type Natriuretic Peptide (NT-proBNP), the New York Heart Association (NYHA) class at 6 months, the change in NYHA when dichotomised into class I/II and III/IV and the 6 min walking distance (6MWD). In the Belgian cohort VO_2_ max was used instead of the 6MWD.

### 2.3. Survival

Our long-term outcome measure was all-cause mortality for the MARC cohort (maximum follow-up period 12 months) and a combined outcome of heart failure hospitalization and all-cause mortality for the Belgian cohort (maximum follow-up period 112 months).

### 2.4. Data Analyses

Continuous data are presented as mean ± standard deviation (SD) when normally distributed, and as median with interquartile range (IQR) when not normally distributed. Categorical data are presented as number (N) and percentages (%). Differences between women and men at baseline and at 6 months follow-up were analyzed using Student T-test, chi-square test, or Mann–Whitney U test, where appropriate. Associations between sex and response measures at 6 months were assessed using linear regression. Variables were log-transformed if not normally distributed. Multivariable adjustment was performed for body surface area and ischemic etiology (classified by medical history of myocardial infarction or revascularization). For the response variable decrease in LVESV >15%, logistic regression was used (yielding Odds Ratio (OR) with 95% confidence intervals (95% CI)). For the entire cohort a forward selection model was used, adding BSA and etiology to sex.

Additionally, the above analyses were repeated in the non-ischemic subgroups of both cohorts. We used Cox regression analysis to assess the association between sex and clinical outcomes. In multivariable Cox regression analyses we adjusted for BSA and etiology. Survival curves are displayed using Kaplan–Meier curves and compared with log-rank testing. Statistical analyses were performed using R.Studio version 3.6.3. A two-tailed *p* value of <0.05 was considered statistically significant.

## 3. Results

### 3.1. Patient Characteristics

In the MARC study, 240 patients were included (39% women), mean age was 66.5 ± 9.7 years, and median LVEF 26 (20–32)%. Baseline characteristics of the MARC cohort are described in Table 1. In brief, women more often had non-ischemic etiology (84% vs. 40% in men), larger inter ventricular mechanical delay (IVMD), more apical rocking (79% vs. 51% in men), and LBBB morphology (74% vs. 52% in men), all *p* values ≤ 0.005. QRS area and duration were similar for both sexes. There was no difference in NT-proBNP or QRS area. Medication did not differ at baseline, except for statin use which was lower in women (40% in women vs. 68% in men, *p* < 0.001). Beta blocker use between women and men did not differ at 6 months follow-up in both the MARC and Belgian cohort (data not shown).

The non-ischemic subgroup of the MARC cohort consisted of 132 patients (55% women). eGFR was significantly lower in non-ischemic women than in non-ischemic men, whereas Galectin-3 levels were higher in women (all *p* ≤ 0.011, Table 2). Similar to the entire cohort, the use of statins was less frequent in women compared to men with non-ischemic etiology (*p* = 0.02). QRS area, QRS morphology, and QRS duration did not differ between the sexes.

In the Belgian cohort (*n* = 818), 32% were women, mean age was 72.0 ± 10.3 years, and median LVEF 30% (25–35)%. Baseline characteristics for the Belgian cohort are shown in Table 1. Women more frequently had a non-ischemic etiology and LBBB pattern, as well as poorer renal function (all *p* values < 0.020).

The non-ischemic subgroup of the Belgian cohort consisted of 468 patients (44% women, Table 2). Frequency of LBBB and QRS duration did not differ between women and men. Creatinine and eGFR was significantly lower in women (all *p* < 0.005).

### 3.2. Response Measures at 6 Months

In the entire MARC cohort, 70% of the women were responders (defined as >15% decrease in LVESV) at 6 months, compared to 55% of the men (*p* = 0.041, Table 3). In univariable analyses, women showed a greater decrease in LVESV (%) and LVESV indexed to BSA (%), compared to men (all *p* < 0.05). Women also significantly improved more in 6MWD and showed a greater decrease in NT-proBNP (all *p* < 0.050). When adjusted for BSA and etiology however, the differences in change in LVESV (%) and LVESV indexed to BSA (%) lost significance. The improvements in 6MWD and NT-proBNP (%) remained significantly greater for women (Table 4). In the non-ischemic subgroup of the MARC cohort, the response measures at 6 months did not differ between women and men (Table 2).

In the Belgian cohort, the number of women who responded was higher (79% vs. 67% of men (*p* = 0.002), Table 3). Similar to the MARC cohort, in the univariable analyses, change in LVEF (%) and LVESV (%) was greater in women than in men (Table 3). Significance for change in LVEF (%) was lost after adjustment for BSA and etiology, but not for change in LVESV (%). Results remained unchanged after additional adjustment for apical rocking (Table 4). For the change in LVESV and LVESVi, the β’s in the multivariable analyses were greater for apical rocking than for ICM (β 12.21 vs. 11.69 for LVESV, and 12.76 vs. 11.66 LVESVi, respectively, all *p* values ≤ 0.001). In the non-ischemic subgroup of the Belgian cohort, women showed a significantly greater decrease in LVESV compared to men, yet no difference was observed for other response measures (Table 2).

Adjustment for height instead of BSA for change in LVEF (%) and LVESV (%) yielded similar findings (data not shown). Also, eGFR declined significantly in the MARC cohort from 71.4 (54–84.9) to 67.6 (55–80.7) (*p* value < 0.001), and remained unchanged in the Belgian cohort (eGFR 59 (41.7–74.7) at baseline and 54 (39–68.3) at 6 months follow-up, *p* value = 0.875).

### 3.3. Sex as Determinant of Response Defined as Decrease in LVESV >15%

When defining response to CRT as a decrease in LVESV >15%, women in the MARC cohort had significantly higher odds of responding compared to men in the univariable analysis (OR (95% CI) 1.92 (1.08–3.49), *p* = 0.030). After adjustment for BSA, and for BSA and etiology combined, this association was no longer significant (Table 4). In the non-ischemic subgroup, women did not have significantly greater odds of responding compared to men (*p* = 0.538).

In the Belgian cohort, women also had higher odds of responding, defined as a decrease in LVESV >15%, in univariable analysis (OR (95% CI) 1.82 (1.26–2.62), *p* = 0.002). Significance remained after adjustment for BSA, but was lost after adjustment for BSA and etiology (Table 4). In the non-ischemic subgroup of the Belgian cohort, sex was not a significant determinant of response (*p* = 0.190).

### 3.4. Survival

**Figure 1:**
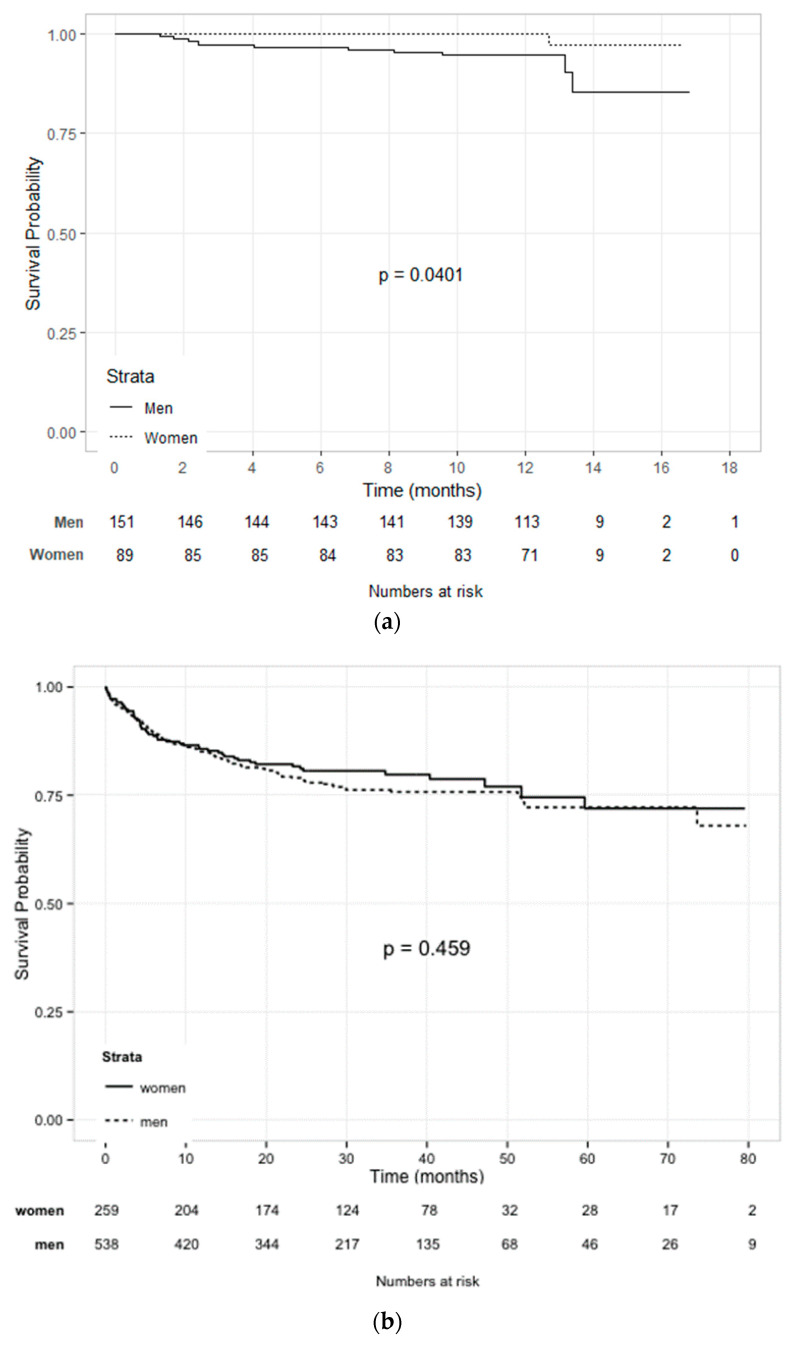
(**a**): Kaplan–Meier for all-cause mortality for groups based on sex in the MARC cohort; (**b**): Kaplan–Meier for mortality and heart failure hospitalization for groups based on sex in the Belgian cohort.

The median (IQR) follow-up in the MARC cohort consisted of 12 (12–13) months. Eleven patients (4.6%) died during follow-up. Survival curves of the MARC cohort are depicted in Figure 1a. Women showed a marginally significant better survival when analyzed using the log-rank test (*p* = 0.04). In univariable Cox regression analyses however, female sex was not associated with a lower risk of death (hazard ratio (HR) (95% CI) 0.19 (0.02–1.50), *p* = 0.115).

During a median (IQR) follow-up of 26 (14–40) months, 168 (21.1%) patients in the Belgian cohort experienced the combined outcome of heart failure hospitalization or all-cause mortality. Survival estimates are depicted in Figure 1B. Survival did not differ between women and men (HR (95% CI) 1.13 (0.81–1.57), *p* = 0.460). Additionally, in the non-ischemic cohort, 64 patients (14%) experienced the combined outcome measure of heart failure hospitalization and all-cause mortality, with no difference in survival between women and men (HR (95% CI) 0.76 (0.47–1.24), *p* = 0.272).

## 4. Discussion

In a large prospective cohort of HFrEF patients receiving CRT implantation for indications according to the guidelines, we investigated sex differences in response to CRT, and sex-specific determinants of response. We used a larger independent retrospective Belgian cohort to validate our findings. Our results show that, compared to men, women are more often responders and show a greater response to CRT as measured by left ventricular remodeling echocardiographic measures, 6MWT, and NT-proBNP. However, this sex difference in LV remodeling response to CRT was lost after accounting for ischemic etiology (classified by medical history of myocardial infarction or revascularization), which confirms our hypothesis that the difference in response is mainly determined by HF etiology, where patients with non-ischemic etiology are more likely to respond to CRT. These findings were confirmed in the independent Belgian cohort. As this study shows this direct relationship between heart failure etiology and reverse remodeling response to CRT in a relatively large Dutch and Belgian cohort, it adds to the evidence in the ongoing discussion that the female sex is very likely not the main determinant for response to CRT.

### 4.1. Sex Differences in Response to CRT

Multiple previous studies have reported a greater benefit of CRT on LV reverse remodeling in women, and suggested female sex as a determinant of response [6,10,16,17]. Our study provides important information on differential response to CRT by sex and expands on current knowledge by studying echocardiographic as well as biochemical response measures following CRT implantation in two independent cohorts. Several types of response have been studied previously, ranging from functional (6MWT, VO2 max, NYHA) to structural changes measured by echocardiographic parameters and survival. In this study, we found that LV remodeling response to CRT was significantly greater in women compared to men. These differences in echocardiographic response between women and men did however not remain after accounting for etiology. Our results stress the importance of etiology as a driver for left ventricular remodeling in HFrEF patients receiving CRT implantation, which has also been suggested by Beela et al. [18]. Previously, Beela et al. reported ischemic HF etiology as the driver for sex difference in volumetric response to CRT. This conclusion was drawn based on the finding of a significant association between dyssynchrony and volumetric response and dyssynchrony and ICM. However, ICM itself was not significantly associated with volumetric response. The current study expands on this suggestion by showing a direct effect of ICM on volumetric response to CRT. Furthermore, our findings are in accordance with findings by Martens et al. showing that LV remodeling was less common in patients with an ischemic etiology. These patients however did derive benefit at a lesser degree of remodeling compared with patients with a non-ischemic etiology, underscoring that the clinical impression of less reverse remodeling based on pre-implant characteristics should not preclude the implantation of a CRT device, as benefits on hard outcome persist [14]. Furthermore, subanalyses of multiple prospective randomized studies, including the Multicenter InSync Randomized Clinical Evaluation (MIRACLE), Cardiac Resynchronization—Heart Failure (CARE-HF) and Multicenter Automatic Defibrillator Implantation with Cardiac Resynchronization Therapy (MADIT-CRT) studies, have confirmed the finding of greater reverse remodeling in non-ischemic cardiomyopathy [16,19,20]. The fibrotic scar tissue in the myocardium after an ischemic event impairs proper conduction of the electrical impulses generated by the CRT device, inhibiting cardiomyocyte contraction and thereby hampering reverse remodeling of the ventricle. Specifically, focal myocardial fibrosis in the vicinity of the LV lead tip leads to reduced response to CRT. In non-ischemic cardiomyopathy, fibrosis may also be present, but its localization is different from ischemic cardiomyopathy, where fibrosis follows subendocardial or transmural distribution along the coronary arteries [21].

### 4.2. Serum Biomarkers, Functional Response and Survival

Unlike the echocardiographic measures, change in NT-proBNP and 6MWT remained significantly greater for women compared to men after adjustment for BSA and etiology, with women showing greater reduction in NT-proBNP and greater improvement in 6MWD. This seemingly contradictory result has been mentioned in previous studies, namely that reverse remodeling (improvement in LVEF) does not necessarily correlate with an improvement in biochemical or clinical response [8,22]. NT-proBNP has been reported previously to correlate with improvements in markers of cardiac function and volume [23]. However, LVESV and LVEF did not show a significant improvement after adjustment, and we were not able to validate the significant improvement in NT-proBNP for women in the Belgian cohort. Also, as the eGFR declined significantly at 6 months follow-up compared to baseline in women in the MARC cohort, the greater reduction in NT-proBNP is unlikely to be caused by an improvement in renal function.

It has recently been suggested that given the discordant results for 6MWT and for instance morbidity and mortality with several treatment options for heart failure, 6MWT might not be the most suitable outcome measure to evaluate response unless the intervention studied is aimed at improving (submaximal) exercise capacity [24]. In the present study we also did not observe a significant difference in survival between the sexes. This finding is in line with several large CRT trials that did not demonstrate a significant interaction between sex and outcomes (HF and mortality) in predefined subgroup analyses [25,26,27]. Although there is a disconnect in response measures observed in women, i.e., no difference in survival yet better LV reverse remodeling, functional and NT-proBNP response, our findings suggest that women with HFrEF receiving CRT could perhaps be a subgroup in which further studies such as into heart failure medication withdrawal may be considered [28]. In patients with a normalized ejection fraction following CRT implantation, it was recently shown that withdrawal of neurohumoral blockers was feasible in 2/3 of patients, yet the ability to withdraw treatment was mainly limited by comorbidities such as hypertension [28]. Sex differences were not taken into consideration in this study. Also, in the current study, we have not provided data on whether this applies to our population as well, and therefore the possibility to withdraw medication should be considered with care.

### 4.3. Determinants of Response to CRT

It is increasingly recognized that women differ from men in several aspects relating to HF therapy. Not only do they show different response to CRT, they are also thought to have different optimal dosages for HF medication [9]. Women generally have a smaller body surface area, which may be a possible driver for the differences regarding HF therapies. Related to this, there is an ongoing discussion whether QRS duration should be individualized to body/heart size, possibly especially influencing CRT indications for women [29,30,31]. The guidelines favor CRT implantation in patients with a broad QRS duration (≥150 ms) and LBBB morphology (class IA recommendation) and do not recommend CRT in patient with QRS duration <120 ms [12]. It has however been reported that in patients with LBBB, women more frequently have true LBBB morphology and more mechanical dyssynchrony at shorter QRS durations compared to men, indicating that also dyssynchrony should be interpreted differently between women and men [32]. Using a sophisticated approach were QRS area was normalized to heart size, using the QRS area/LVEDV ratio, Salden and colleagues showed that women have a larger QRS area/LVEDV ratio and this contributes to a greater change in LVESV in women after CRT [33]. This association remained significant after adjusting for, among other things, ischemic etiology (classified by medical history of myocardial infarction or revascularization). Based on this, it is suggested that greater electrical dyssynchrony in smaller hearts is responsible for the improved reverse remodeling response in women. In our study, we were only able to replicate this analysis in the MARC cohort, showing independent associations with LV remodeling response for QRS area/LVEDV ratio and ischemic etiology, however not for female sex (data not shown). It is possible that our study is underpowered for these analyses, however it confirms the finding that other contributory factors, possibly inherent to the female sex, are responsible for the greater LV remodeling response in women. Also, in the multivariable analyses in the MARC cohort, apical rocking had a slightly higher β compared to ICM for response measured by LVESV, but not for other measures of response. This finding suggests that apical rocking would have a greater influence on the response to CRT measured by LVESV than ICM. Apical rocking has previously also been reported to as a predictor of clinical and echocardiographic response to CRT [34]. However, this finding could only be shown in the MARC cohort. This result could not be validated as there was no data on apical rocking available in the Belgian cohort. Nevertheless, apical rocking seems to be another valuable tool to use in the prediction of response to CRT. Finally, despite the repeated finding that response to CRT is more pronounced in women, CRT is still underused in women representing only 24% of implantations in ESC survey II [35]. Our data underscores the importance of CRT implantation in women as we demonstrated that remodeling response is generally better and outcomes are comparable. In both cohorts only 32–39% of patients were women, also illustrating the possible underuse of CRT in women.

#### 4.3.1. Strengths and Limitations

The MARC study is a multicenter effort and the only study to date which prospectively studied the effect of biomarkers on CRT response, and our results have been validated in a large Belgian retrospective cohort. A limitation of this study was that we could not validate our findings of 6MWT and NT-proBNP in the Belgian cohort. However, we did use VO2 max, which is the gold standard for assessing maximal exercise capacity. Furthermore, the long-term outcome measure of the MARC cohort consisted of mortality while this was mortality combined with HF hospitalization in the Belgian cohort. Unfortunately, there was no data available on the number of hospitalizations in the MARC cohort. Furthermore, we do not have non-invasive data on ischemia available and classified patients as having ICM based on their medical history of myocardial infarction or revascularization. Although this definition of ICM is used more often, it would have been more favorable to base this classification on imaging data showing myocardial scarring. Finally, our subgroup analyses in the non-ischemic cohort, particularly in the MARC study, might have been underpowered to detect significant differences. Therefore, these analyses should be replicated in even larger cohorts in future studies.

#### 4.3.2. Future Steps

Our study indeed demonstrates that women show a greater response to CRT in terms of LV reverse remodeling, which is most likely due to the higher prevalence of non-ischemic cardiomyopathy. However, this does not mean that CRT would not benefit men with ischemic cardiomyopathy. As our results show, and the study by Martens et al. supports, risk of outcome is similar for women and men, independent of their HF etiology and despite differences in LV remodeling [14]. Furthermore, our results imply that, in order to attain an improved prediction of response to CRT, not only HF etiology should be considered, but also the electrical dyssynchrony. Several measures of electrical dyssynchrony are being used in studies and clinical practice such as QRS area, QRS duration, QRS morphology, apical rocking, and most recently QRS area/LVEDV ratio where electrical dyssynchrony is indexed to heart size. QRS index is another measure which may be considered to identify responders to CRT [36,37]. Future studies, using large cohorts, should focus on which measure is most informative for the prediction of CRT response in ischemic and non-ischemic etiology and possibly take into account individualized measures of electrical dyssynchrony adjusted for body/heart size.

## 5. Conclusions

In conclusion, this study shows that women with HFrEF respond better to CRT in terms of LV reverse remodeling compared to men, significance was however lost after adjustment of ischemic etiology. Our findings suggest that non-ischemic etiology, rather than female sex, is responsible for greater response rates in women treated with CRT compared to men.

## Figures and Tables

**Table 1:** Baseline characteristics based on sex.

	MARC Cohort			Belgian Cohort		
Patient Characteristics	Women (*n* = 87)	Men (*n* = 146)	*p* Value	Women (*n* = 262)	Men (*n* = 556)	*p* Value
Age (years)	64.9 ± 10.4	67.5 ± 9.1	0.050	72.3 ± 10.2	71.7 ± 10.3	0.390
BSA (m^2^)	1.8 ± 0.2	2 ± 0.2	<0.001	1.7 (1.6–1.9)	1.9 (1.8–2)	<0.001
Height (cm)	165.9 ± 7.7	177.3 ± 7.8	<0.001	161.3 ± 8.5	171.6 ± 7.9	<0.001
Weight (kg)	74.8 ± 14.3	86.2 ± 16.8	<0.001	70 ± 13.9	79.8 ± 15.6	<0.001
Comorbidities						
DM, *n* (%)	18 (21%)	45 (31%)	0.126	66 (25%)	151 (27%)	0.600
Atrial fibrillation, *n* (%)	9 (10%)	23 (16%)	0.335	91 (35%)	212 (38%)	0.369
Cardiomyopathy						
Non-ischemic	73 (84%)	59 (40%)	<0.001	205 (78%)	263 (48%)	<0.001
eGFR (ml/min/1.73 m^2^)	71.4 (54–84.9)	69.8 (53.6–89.9)	0.509	59 (41.74.7)	64.3 (46.7–79.5)	0.008
NYHA class	II (II–III)	II (II–III)	0.304	III (II–III)	III (II–III)	0.341
6-MWD (m)	379.9 ± 104.2	427.7 ± 102.6	0.002			
VO_2_ max				13.1 (11.2–15.7)	14.7 (11.9–19)	<0.001
VCG and echocardiography						
QRSarea (µVs)	135.3 ± 37.5	130.5 ± 52.4	0.445			
Inter-ventricular mechanical delay (ms)	53.9 ± 23.3	41.8 ± 30.8	0.001			
Apical rocking present	68 (79%)	74 (51%)	<0.001			
Serum biomarkers						
NT-proBNP (ng/L)	1063 (412–1997)	932 (440–1765)	0.299			
hsTroponin T (ng/L)	21 (11–28)	24 (16–33)	0.010			
Aldosterone (pg/mL)	54.6 (33–105.1)	73.9 (32.3–141.2)	0.302			
Galectin 3 (ng/mL)	18.6 (14.7–22.7)	16.1 (13.2–20.6)	0.049			
Creatinine (µmol/L and mg/dL)	76 (65–93)	94 (77–117)	<0.001	88 (71–115)	106 (88–133)	<0.001
Ureum (mmol/L)	7 (5.4–9.3)	7.6 (6.3–9.8)	0.066			
LV ejection fraction (%)	27 (20–32)	25 (20–31)	0.336	30 (25–35)	30 (25–35)	0.907
LVESV (mL)	117 (89–147.3)	144 (115.8–187.4)	<0.001	110.6 (80.9–152.2)	124.9 (90.7–162)	0.021
LVESV index (mL/m^2^)	64.6 (49.4–82.2)	71.1 (56.3–91.6)	0.064	67.4 (45.3–89.2)	65.1 (47.2–88.4)	0.813
QRS morphology						
LBBB	61 (74%)	73 (52%)	0.005	211 (81%)	394 (73%)	0.011
QRS duration						
Continuous	178 (166–189)	179 (162–194)	0.765	149 (134–164)	156 (130–174)	0.019
≥150 ms	76 (93%)	124 (91%)	0.761	148 (57%)	337 (62%)	0.188
Medication						
Beta blocker	80 (92%)	123 (84%)	0.134	222 (85%)	453 (82%)	0.458
ACE-inhibitor or ARB	83 (95%)	136 (93%)	0.678	229 (87%)	457 (83%)	0.138
Aldosterone antagonist	44 (51%)	69 (47%)	0.723	175 (67%)	335(61%)	0.123
Loop diuretic	61 (70%)	96 (66%)	0.588	129 (49%)	254 (46%)	0.487
Other diuretic	5 (6%)	10 (7%)	0.956	27 (20%)	62 (11%)	0.764
Statin	35 (40%)	100 (68%)	<0.001	117 (45%)	346 (63%)	<0.001

Abbreviations: 6MWD: 6 min walking distance; ACE-inhibitor: angiotensin converting enzyme inhibitor; ARB: angiotensin receptor blocker; BSA: body surface area; DM: diabetes mellitus; eGFR: estimated glomerular filtration rate; GDF15: growth/differentiation factor 15; hsCRP: high-sensitive C-reactive protein; IL6: interleukin-6; LBBB: left bundle branch block; LVEF: left ventricular ejection fraction; LVESV: left ventricular end systolic volume; NT-proBNP: N-terminal B-type natriuretic peptide; NYHA: New York Heart Association; VCG: vectorcardiography.

**Table 2:** Baseline table and response measures at 6 months follow-up of patients with non-ischemic etiology.

	MARC Cohort			Belgian Cohort		
Patient Characteristics	Women (*n* = 73)	Men (*n* = 59)	*p* Value	Women (*n* = 205)	Men (*n* = 263)	*p* Value
Age (years)	64.2 ± 10.4	63.4 ± 9.8	0.660	71.8 ± 10.4	69.3 ± 11.6	0.016
BSA (m^2^)	1.8 ± 0.2	2.1 ± 0.2	<0.001	1.7 (1.6–1.9)	1.9 (1.8–2.1)	<0.001
Comorbidities						
DM, *n* (%)	15 (21%)	15 (25%)	0.649	44 (22%)	48 (18%)	0.453
Atrial fibrillation, *n* (%)	7 (10%)	10 (17%)	0.320	68 (33%)	101 (38%)	0.284
eGFR (ml/min/1.73 m^2^)	71.9 (55.3–86.6)	85 (63.4–96.9)	0.011	63 (43.4–75.9)	68 (53.7–83.1)	0.003
NYHA class	II (II–III)	II (II–III)	0.174	III (II–III)	III (II–III)	0.160
6-MWD (m)	387 ± 105	446 ± 95	0.002			
VO2 max				12.9 (11–15.7)	15.5 (12.6–19.1)	<0.001
VCG and echocardiography						
QRSarea (µVs)	136.4 ± 37.7	149.1 ± 49.2	0.129			
Inter-ventricular mechanical delay (ms)	54.3 ± 23.1	57.6 ± 27.4	0.478			
Apical rocking present	59 (81%)	40 (68%)	0.129			
Serum biomarkers						
NT-proBNP (ng/L)	1031 (485–1954)	826 (358–1750)	0.164			
LV ejection fraction (%)	26 (20–32)	25 (20–31)	0.471	30 (25–35)	30 (25–35)	0.900
LVESV (mL)	117 (89–147.3)	140.7 (117.8–186)	0.019	110.6 (80.9–152.2)	121.5 (91.9–157.7)	0.060
LVESV indexed to BSA (mL/m^2^)	63.6 (49.4–82.2)	70.8 (54.8–86.6)	0.290	66.5 (44.8–89.2)	63.2 (47.4–86.4)	0.738
QRS morphology						
LBBB	53 (76%)	39 (72%)	0.403	169 (83%)	199 (77%)	0.113
QRS duration						
Continuous	178 (166–189)	180 (168–194)	0.293	150 (134–164)	156 (132–174)	0.072
≥150 ms	63 (91%)	53 (93%)	0.987	118 (58%)	173 (67%)	0.069
Medication						
Beta blocker	67 (92%)	51 (86%)	0.480	174 (85%)	211 (81%)	0.309
ACE-inhibitor or ARB	70 (96%)	59 (100%)	0.323	179 (87%)	220 (84%)	0.429
Aldosterone antagonist	34 (47%)	29 (49%)	0.905	135 (66%)	156 (60%)	0.211
Loop diuretic	52 (71%)	38 (64%)	0.516	101 (49%)	103 (40%)	0.047
Other diuretic	3 (4%)	5 (9%)	0.498	21 (10%)	29 (11%)	0.881
Statin	25 (34%)	33 (56%)	0.020	75 (37%)	124 (48%)	0.026
Response measures at 6 months follow-up						
LVEF at 6 months	37 (28–43)	34 (26–39)	0.248	50 (40–50)	45 (40–55)	0.467
Absolute change	11.6 (3.1–14.5)	9.5 (1.9–14.8)	0.841	15.5 (5–25)	15 (5–25)	0.278
% change	36.9 (13.5–60.7)	35.4 (6–67)	0.998	60 (17.5–100)	52.6 (19.2–100)	0.318
LVESV, ml at 6 months	77 (50–98.3)	95.5 (66.5–142.7)	0.016	63.3 (46.1–85.2)	73.7 (58.3–99.0)	<0.001
Absolute change	−35.3 (−57.3–−15)	−34.5 (−70–−13.9)	0.625	−42.3 (−76.6–−19.1)	−44.6 (−81.2–−17.7)	0.794
% change	−36.5 (−45.3–−12.4)	−27.2 (−45.8–−12.9)	0.570	−44.2 (−59.4–−26.6)	−38.9 (−53.3–−22.3)	0.028
>15% decrease, *n* (%)	51 (74%)	40 (69%)	0.675	148 (81%)	177 (76%)	0.233
LVESV indexed to BSA at 6 months	40.6 (28.4–53.7)	44.5 (34.7–65.9)	0.229	36.1 (26.3–47.9)	37.7 (28.6–53.9)	0.116
% change	−36.4 (−44.4–−10.8)	−27.2 (−45.5–−14.1)	0.596	−41.5 (−58.3–−22.8)	−38.3 (−53.2–−17.3)	0.219
6MWD, m	450 (372–496)	500 (430–545)	0.004			
Absolute change	38.5 (16.8–83)	33.5 (0–57.8)	0.135			
% change	8.7 (4–20.7)	7.4 (0–13.2)	0.065			
VO2 max absolute change				0 (−1.4–2.6)	1.2 (1.2–3.8)	0.131
NT-proBNP, ng/mL	444 (157.5–853)	321 (126–586)	0.299			
Absolute change	−471 (−1072–−161)	−237 (−762–−24)	0.037			
% change	−62 (−79.7–−28.7)	−58.8 (−71.5–−14.3)	0.272			
NYHA at 6 months	II (II–II)	II (I–II)	0.069	II (I–II)	I (I–II)	0.016
NYHA change *, *n* (%)	0 (−1–1)	0 (0–0)	0.361	−1 (−1–0)	−1 (−2–−1)	0.221

* NYHA dichotomised into class I/II and class III/IV. Abbreviations: 6MWD: 6 min walking distance; ACE-inhibitor: angiotensin converting enzyme inhibitor; ARB: angiotensin receptor blocker; BSA: body surface area; DM: diabetes mellitus; eGFR: estimated glomerular filtration rate; GDF15: growth/differentiation factor 15; hsCRP: high-sensitive C-reactive protein; IL6: interleukin-6; LBBB: left bundle branch block; LVEF: left ventricular ejection fraction; LVESV: left ventricular end systolic volume; NT-proBNP: N-terminal B-type natriuretic peptide; NYHA: New York Heart Association; VCG: vectorcardiography.

**Table 3:** Response measures at 6 months follow-up.

	MARC Cohort			Belgian Cohort		
Outcome Measures	Women (*n* = 87)	Men (*n* = 146)	*p* Value	Women (*n* = 262)	Men (*n* = 556)	*p* Value
LVEF	36 (25–43)	32 (24–37)	0.009	45 (40–55)	43 (35–50)	<0.001
Absolute change	7 (2–14)	6 (0–12)	0.063	15 (5–25)	10 (5–20)	0.001
% change	29.6 (5.7–60.1)	22.2 (0.3–54.5)	0.160	50.0 (14.3–100.0)	37.5 (12.5–76.2)	0.006
LVESV	77 (57–111.7)	114.8 (79.8–151.3)	<0.001	64.8 (46.2–88.5)	84.2 (60.9–122.5)	<0.001
Absolute change	−32.7 (−57–−11)	−23.8 (−54.4–−3.4)	0.247	−39.6 (−70.3–−14.9)	−36.0 (−46.9–−12.6)	0.101
% change	−33 (−41.9–−9.4)	−18 (−37.2–−2.4)	0.014	−40.8 (−56.0–−23.0)	−30.8 (−46.9–−12.6)	<0.001
>15% decrease, *n* (%)	57 (70%)	73 (55%)	0.041	184 (79%)	326 (67%)	0.002
LVESV indexed to BSA	41.9 (32.7–57.1)	55.7 (40.1–73.3)	<0.001	36.1 (27.6–49.4)	43.9 (30.9–66.3)	<0.001
% change	−34.1 (−43.7–−9)	−19 (−36.9–−1.4)	0.012	−39.05 (−54.5–−18.5)	−29.8 (−46.6–−10.5)	<0.001
6MWD	443 (371–492)	469 (409–529)	0.030			
Absolute change	39.5 (18.8–86.8)	31 (0.5–61.5)	0.042			
% change	9 (4.5–21)	7.1 (0.1–14.6)	0.016			
VO2 max absolute change				0 (−1.3–2.5)	0.8 (−1.4–3.2)	0.320
NT-proBNP	532 (157–1119.5)	552 (262.2–1348.2)	0.217			
Absolute change	−430 (−1072–−151.5)	−186 (−598.5–96)4	<0.001			
% change	−57.9 (−78.5–−19.5)	−31.4 (−60.3–20.3)	<0.001			
NYHA at 6 months	II (II–II)	II (II–II)	0.578	II (I–II)	II (I–II)	0.031
NYHA change *, *n* (%)	0 (−1–0)	0 (0–0)	0.350	−1 (−1–0)	−1 (−1–0)	0.249

* NYHA dichotomized into class I/II and class III/IV. Abbreviations: 6MWD: 6 min walking distance; BSA: body surface area; LVEF: left ventricular ejection fraction; LVESV: left ventricular end systolic volume; NT-proBNP: N-terminal B-type natriuretic peptide; NYHA: New York Heart Association; SE: standard error.

**Table 4:** Association of sex and response measures at 6 months follow-up.

	MARC Cohort								Belgian Cohort					
Adjusted for	Unadjusted		BSA		BSA and Etiology		BSA, Etiology and Apical Rocking		Unadjusted		BSA		BSA And Etiology	
Response Measures	*β*	*p* Value	*β*	*p* Value	*β*	*p* Value	*β*	*p* Value	*β*	*p* Value	*β*	*p* Value	*β*	*p* Value
LVEF, %														
% change	10.373	0.112	9.894	0.193	−0.027	0.997	−1.361	0.867	3.141	0.001	3.213	0.003	1.061	0.324
LVESV, mL														
% change	−7.875	0.021	−6.987	0.071	−0.337	0.933	1.358	0.731	−9.538	<0.001	−9.803	<0.001	−5.660	0.036
LVESV indexed to BSA % change **	8.033	0.020			2.382	0.505	0.094	0.979	−7.529	0.001			−3.886	0.105
6MWD, m														
Absolute change	20.75	0.031	26.92	0.016	27.55	0.024	23.211	0.063						
% change, log10	0.034	0.032	0.040	0.032	0.050	0.014	0.042	0.044						
NT-proBNP, ng/mL														
Absolute change	−399.2	0.062	−485.4	0.043	−329	0.207	−253.4	0.332						
% change, log10	−0.223	<0.001	−0.254	<0.001	−0.137	0.048	−0.111	0.105						
NYHA change	−0.032	0.632	−0.067	0.369	−0.059	0.470	−0.058	0.481	0.065	0.254	0.033	0.598	0.062	0.342
**Adjusted for**	**OR (CI)**	***p*** **Value**	**OR (CI)**	***p*** **Value**	**OR (CI)**	***p*** **Value**	**OR (CI)**	***p*** **Value**	**OR (CI)**	***p*** **Value**	**OR (CI)**	***p*** **Value**	**OR (CI)**	***p*** **Value**
LVESV >15% decrease	1.92 (1.08–3.49)	0.030	1.92 (0.99–3.75)	0.054	1.28 (0.60–2.59)	0.559	1.07 (0.50–2.28)	0.852	1.82 (1.26–2.62)	0.002	1.85 (1.25–2.76)	0.002	1.45 (0.96–2.20)	0.079

** Only adjusted for etiology. Abbreviations: 6MWD: 6 min walking distance; BSA: body surface area; CI: Confidence Interval; LVEF: left ventricular ejection fraction; LVESV: left ventricular end systolic volume; NT-proBNP: N-terminal B-type natriuretic peptide; NYHA: New York Heart Association; OR: Odds Ratio; SE: standard error.

## Abbreviations

6MWD/6MWT	6 min walking distance/6 min walking test
ACE-inhibitor	Angiotensin converting enzyme inhibitor
ARB	Angiotensin receptor blocker
BSA	Body surface area
CRT	Cardiac resynchronization therapy
DM	Diabetes mellitus
eGFR	Estimated glomerular filtration rate
HFrEF	Heart failure with reduced ejection fraction
IVCD	Interventricular conduction delay
LBBB	Left bundle branch block
LVEF	Left ventricular ejection fraction
LVESV	Left ventricular end systolic volume
MARC study	Markers and Response to CRT study
NT-proBNP	N-terminal pro B-type natriuretic peptide
NYHA	New York Heart Association
VCG	Vector cardiography
